# Simple Approach to Enhance Green Tea Epigallocatechin Gallate Stability in Aqueous Solutions and Bioavailability: Experimental and Theoretical Characterizations

**DOI:** 10.3390/ph14121242

**Published:** 2021-11-30

**Authors:** Philippe-Henri Secretan, Olivier Thirion, Hassane Sadou Yayé, Thibaud Damy, Alain Astier, Muriel Paul, Bernard Do

**Affiliations:** 1 Matériaux et Santé, Université Paris-Saclay, 92296 Châtenay-Malabry, France; bernard.do@aphp.fr; 2 Department of Pharmacy, Hôpitaux Universitaires Henri Mondor, AP-HP, 94000 Créteil, France; olivier.thirion@aphp.fr (O.T.); prof.astier@gmail.com (A.A.); muriel.paul@aphp.fr (M.P.); 3 Department of Pharmacy, Hôpitaux Universitaires Pitié-Salpêtrière, AP-HP, 75013 Paris, France; hassane.sadou-yaye@aphp.fr; 4 Département de Cardiologie et des Maladies Vasculaires, Hôpitaux Universitaires Henri Mondor, AP-HP, 94000 Créteil, France; thibaud.damy@aphp.fr; 5 EpidermE, Université Paris Est Creteil, 94010 Creteil, France

**Keywords:** epigallocatechin gallate, formulation, complex, stability, mass spectrometry, density functional theory, oral solution

## Abstract

Because of its antioxidant, antimutagenic, and anti-infectious properties, epigallocatechin gallate (EGCG) is the most interesting compound among the green tea catechins polyphenols. However, its health effects are inconclusive due to its very low bioavailability, largely due to a particular instability that does not allow EGCG to reach the potency required for clinical developments. Over the last decade, many efforts have been made to improve the stability and bioavailability of EGCG using complex delivery systems such as nanotechnology, but these efforts have not been successful and easy to translate to industrial use. To meet the needs of a large-scale clinical trial requiring EGCG in a concentrated solution to anticipate swallowing impairments, we developed an EGCG-based aqueous solution in the simplest way while trying to circumvent EGCG instability. The solution was thoroughly characterized to sort out the unexpected stability outcome by combining experimental (HPLC-UV-mass spectrometry and infrared spectroscopy) and computational (density functional theory) studies. Against all odds, the EGCG–sucrose complex under certain conditions may have prevented EGCG from degradation in aqueous media. Indeed, in agreement with the ICH guidelines, the formulated solution was shown to be stable up to at least 24 months under 2–8 °C and at ambient temperature. Furthermore, considerable improvement in bioavailability in rats, against EGCG powder formulated in hard-gel capsules, was shown after gavage. Thus, the proposed formulation may provide an easily implementable platform to administer EGCG in the context of clinical development.

## 1. Introduction

Epigallocatechin gallate (EGCG), by far the most abundant of green tea catechin polyphenols, has been recognized for its antioxidant [1] and antiproliferative [2] effects. Thus, either alone or in combination with conventional therapeutics, EGCG may be used to reduce oxidative damage to lipids, proteins, and DNA [3] or to prevent tumor progression [4]. However, because of its high instability, relative low solubility in water, low intestinal permeability, and short plasma half-life, EGCG exhibits poor biopharmaceutical properties, with very low bioavailability after oral consumption or administration (≤10%), thus resulting in plasma concentrations up to 50 times less than those needed to exert biological activities in in vitro systems [5]. Therefore, up until now, being able to harness the properties of EGCG for therapeutic purposes has been far from conclusive. Only one ointment-based specialty used in the treatment of genital warts is on the market with a short shelf life (Veregen^®^). As of the 11 November 2021, 90 studies are registered in clinical trials.gov. Among these, nine are recruiting patients whose conditions are very different from one clinical trial to the other as the patients suffer from cancer, obesity, or cirrhosis. The typical EGCG regimens consist of 400 mg to 800 mg per day administered per os.

From the formulator’s point of view, the EGCG challenge is highly complicated. Indeed, its high antioxidant properties make it particularly susceptible to oxidative degradation, which requires consideration of pH [6], reducing oxygen content during the manufacturing process and in the finished product [7], adding antioxidant agents while knowing that most of them are less reducing than EGCG and thus offer only low protection, and keeping metal ions (iron, zinc, copper, aluminum) as low as possible [8]. Thus, EGCG is very quickly degraded into GCG by epimerization, quinone derivatives, dimer quinone, and EGCG–EGCG quinone dimers [9]. Aside from GCG and some dimers (theacitrins, theaflavins, theasinensins), which exhibit similar biological activities to those of EGCG, quinone-derived products are to be maintained to concentrations as low as possible as quinones are reported to induce toxic effects at low concentrations. In that sense, stabilization of EGCG is important both to keep the drug’s potency to its intended level and to limit safety issues.

Several technologies have been proposed to improve the solubility and stability of EGCG. Numerous approaches employing encapsulation systems have been described [8], and more recently, other strategies using nanostructure-based drug delivery systems, molecular modification, and co-administration of catechins with other bioactives have also been tested [10]. While some of these concepts have contributed to a better understanding of certain mechanistic aspects, the obstacles to scaling up are significant in terms of manufacturing and the value of using natural, non-transformed ingredients may be lost [11].

This is why, in this area, there is still room for improvement, and on the occasion of a request for a clinical trial, we have proposed a simple formulation, easily controllable and expertly evaluated by health authorities, based on an oral solution to ease the administration of the product to subjects participating in the biomedical research with swallowing impairments or difficulties [12]. This development was based on principles directly related to the intrinsic behaviors of EGCG, and the system was thoroughly characterized by experimental and ab initio density functional theory (DFT)-based approaches. Its performance has also been evaluated by stability studies under stress conditions and long-term studies in accordance with ICH, and by a comparative bioavailability study in rats against powdered EGCG capsules like most commercial products sold as dietary supplements.

## 2. Results and Discussion

### 2.1. Approach to Stabilize EGCG in Aqueous Solution

It is well known that EGCG degradation in aqueous solutions mainly proceeds through oxidation and autoxidation involving several factors, among which the most influencing are pH, light, heat, dissolved oxygen, and metal ions [13,14,15,16]. As a result, the formulation consisted of stabilizing the EGCG liquid dosage form by using suitable excipients and controlling some process parameters to target these main factors responsible for catechins degradation.

Thus, the composition of the formulation, packed in a 250 mL multiple-dose amber glass bottle (Type III) with a polyethylene cap with a polyethylene seal, is reported in Table 1 and an example of the manufacturing process is provided in the Materials and Methods section. Citric acid was used to fix the pH of the oral solution to 3.5–4.5, a range optimal for EGCG stability. Indeed, at higher pH (pH > 5), a small portion of the polyphenol can lose a proton, leading to electron capture, resulting in radical formation of reactive species. Besides, as metal ions may catalyze EGCG degradation, the presence of citric acid is also meant to scavenge these potential trace impurities by formation of complexes. Glucose was used as a reducing sugar and a radical scavenger. Sucrose, on the other hand, does not play a direct role in the stabilization of catechins, mechanistically speaking. In fact, the aim was to substantially improve the solubility of EGCG, resulting in a much more concentrated solution of the active ingredient, and as far as EGCG is concerned, this was clearly in favor of its stability. Indeed, this stabilization strategy was based on the fact that concentrated EGCG is more susceptible to reversible epimerization than irreversible oxidation and autoxidation, according to published data [17,18], and the results of our work presented below (see Section 2.3. Stability of the oral formulation). EGCG in purified and decaffeinated green tea extract (>98% *w*/*w*) is naturally much less soluble in water than EGCG in non-purified green tea extract. The presence of other polyphenols, amino acids, and caffeine highly contributes to its solubility. However, for quality control reasons and especially because of the cardiac profile of the patients involved in the clinical trial, only purified and decaffeinated green tea extract was used in the clinical formulation. In this case, we found that EGCG without sucrose but in the presence of the other excipients is soluble up to a concentration of about 4.6 mg·mL^−1^ at 25 °C (i.e., EGCG was slightly soluble as per the Ph. Eur.). In the formulation presented in Table 1, a solution with an EGCG concentration of 26.7 mg·mL^−1^ was obtained at 25 °C (i.e., in the formulation, EGCG was sparingly soluble as per the Ph. Eur.), an improvement of about a factor of 6.

Regarding sucrose content, the clinical trial planned to administer EGCG in two doses of 400 mg per day in most cases, which, according to our formulation, would provide 7 g glucose and 3 g sucrose. This sugar intake is no greater than that provided by conventional syrup-based treatments such as doxycycline and acetaminophen [19].

Below are the studies showing interactions between EGCG and sucrose that may provide plausible explanations for such results.

### 2.2. Characterization of the Phenomena Involved in the Formula

**Figure 1:**
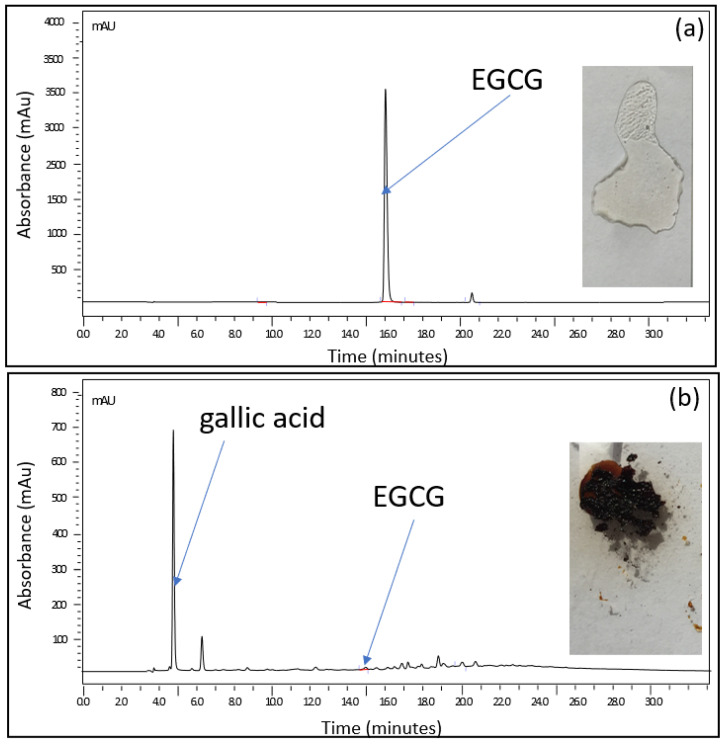
Stability of EGCG versus that of equimolar EGCG/sucrose; 1 mL of (**a**) an equimolar EGCG/sucrose solution (EGCG = 25 mg·mL^−1^; sucrose = 19 mg·mL^−1^) and (**b**) an EGCG-based solution alone (25 mg·mL^−1^) subjected to a thermal gradient program ranging from 25 to 115 °C at a rate of 5 °C per min. At the end of this exposure, part of each of the residues formed was deposited on a glass slide and the remaining part was analyzed using high-performance liquid chromatography.

Complexation of drugs is considered one of the most suitable processes to enhance the stability as well as the aqueous solubility of hydrophobic drugs [20]. Therefore, the search for water-soluble organic ligands that could form complexes with EGCG was central to our formulation process. A screening of ligands of carbohydrates was carried out by investigating the physical change of EGCG solutions spiked with equimolar concentrations of trehalose, sucrose, lactose, or glucose. A simple test was set up for this purpose: 1 mL of an EGCG-based solution alone and a solution/suspension containing one of those ligands were subjected to a thermal gradient program ranging from 25 to 115 °C at a rate of 5 °C per min. At the end of this exposure, part of each of the residues formed was deposited on a glass slide and the remaining part was analyzed using high-performance liquid chromatography. Figure 1 shows the results obtained in the presence of sucrose. The two solutions were initially clear and transparent. When heated, the residue of the EGCG-based solution alone turned black, while a transparent glassy film was observed in place of the EGCG–sucrose solution. When subjected to analysis by chromatography, the black residue contains practically no more EGCG, whereas the chromatographic profile of the product resulting from the heat treatment of the formulation tested is identical to that of the starting solution (Figure 1).

We found out that among the ligands tested, only sucrose or trehalose in combination with EGCG at molar ratio 1:1 has the capacity to protect EGCG. These initial results were a forerunner of what could happen in the formulation.

As sucrose is the most used sweetener by the pharmaceutical industry [21], it was thereafter chosen for the formulation.

#### 2.2.1. Experimental Studies of the EGCG–Sucrose Complex

**Figure 2:**
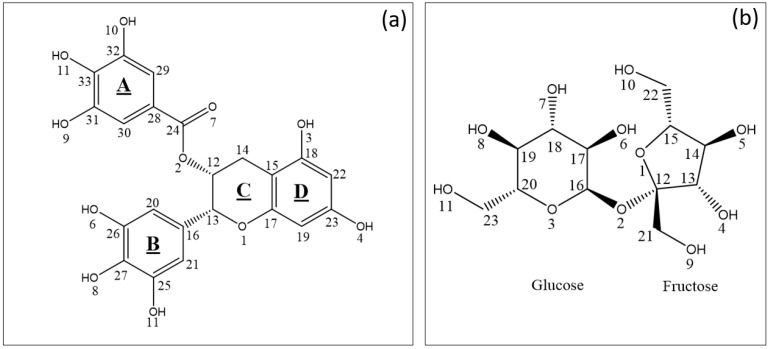
(**a**) Structure and arbitrary numbering of EGCG. (**b**) Structure and arbitrary numbering of sucrose (inset b).

To highlight the possible role of sucrose towards the stabilization of EGCG (Figure 2) in solution, attenuated total reflection Fourier transform infrared spectroscopy (ATR-FTIR) and mass spectrometry (MS) were used to highlight the interaction of EGCG with its ligand and the stoichiometry of the complex. Further, theorical chemistry approaches based on the density functional theory (DFT) were used to determine the structures of the main complexes that may be formed in water and compare their solubility to that of EGCG and sucrose (see Section 2.2.2. Theoretical studies (DFT) of the EGCG–sucrose complex).

##### ATR-FTIR Analysis

**Figure 3:**
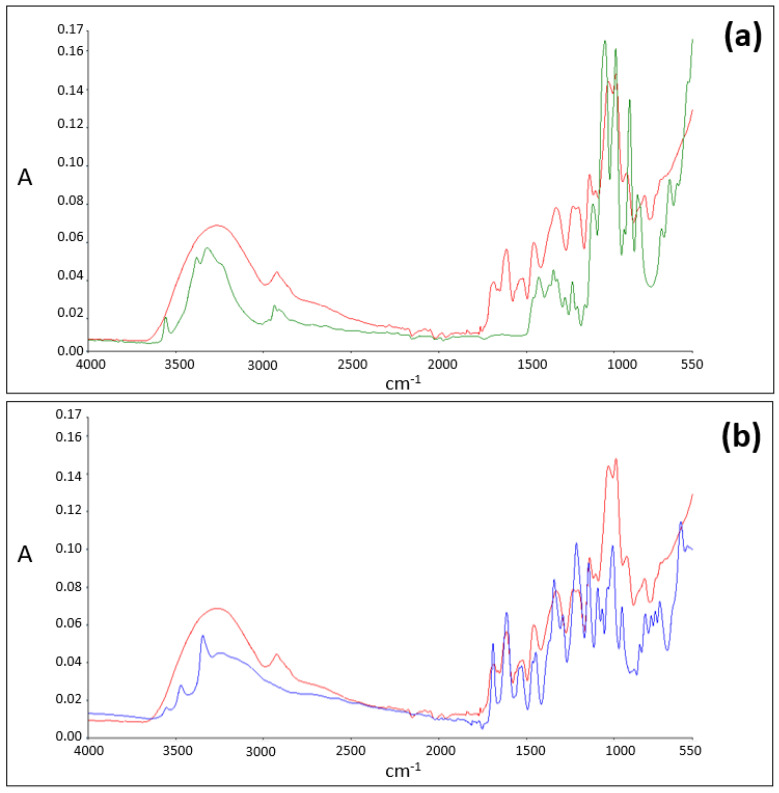
(**a**) ATR-FTIR spectra of sucrose (in green) and of the EGCG–sucrose complex (in red). (**b**) ATR-FTIR spectra of EGCG (in blue) and of the EGCG–sucrose complex (in red).

For sucrose (Figure 3, spectrum in green) and EGCG (Figure 3, spectrum in blue) alone, O-H free stretch peaks are detected in the 3200–3600 cm^−1^ region of the spectra. However, when an equimolar blend of EGCG and sucrose is analyzed, in the same region of the spectrum, the presence of broad absorption O-H stretch IR regions and the absence of O-H free stretch peaks is noticed (Figure 3, spectrum in red). 

These results suggest that EGCG (Figure 2a) and sucrose (Figure 2b) form a complex where H-bonding is involved.

##### Mass Spectrometry Analysis

Electrospray ionization mass spectrometry (ESI-MS) is a powerful means of studying non-covalent complexes between ‘‘host” and “guest’’ with high sensitivity and rapidity [22,23,24,25]. Therefore, to study the stoichiometry of the EGCG–sucrose complex, an aqueous solution containing dissolved EGCG (10 mg·mL^−1^) and sucrose (50 mg·mL^−1^) was extemporaneously diluted a 100-fold in water and directly infused by a syringe pump in the electrospray source (ESI) of the mass spectrometer. 

**Figure 4:**
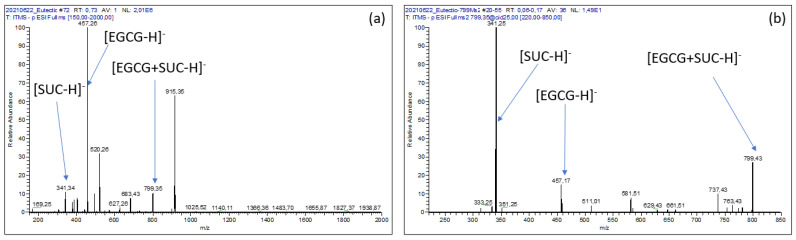
(**a**): ESI^-^ MS spectrum of an EGCG–sucrose solution; (**b**): ESI^-^ MS^2^ spectrum of the [EGCG + SUC − H]^−^ product ion (*m*/*z* = 799).

MS analysis led to the detection of not only the deprotonated ion of EGCG ([EGCG − H]^−^; *m*/*z* = 457) and that of sucrose ([SUC − H]^−^; *m*/*z* = 341) with relative abundances of about 100% and 10%, respectively (Figure 4a), but also an ion imputable to the equimolecular EGCG–sucrose complex ([EGCG + SUC − H]^−^; *m*/*z* = 799). The latter could have been formed as adduct within the ESI source (Figure 4b). However, this signal tends to fade relative to those of EGCG and sucrose when more dilute solutions were analyzed, suggesting that the putative adduct is more likely to have originated from the EGCG–sucrose complex in the solution than from in situ formation at the source.

#### 2.2.2. Theoretical Studies (DFT) of the EGCG–Sucrose Complex

##### Possible Interactions Sites of EGCG and Sucrose

Based on EGCG and sucrose structures (Figure 2), numerous hydrogen bonds OH–O can be formed. In order to assess which equimolar complexes are likely to prevail, the molecular electrostatic potential (MEP) of the atoms were compared for both molecules as MEP has been shown to be a good predictor of the molecular sites likely to interact with other molecules [26,27].

As far as EGCG is concerned, the lowest MEP values (global mean = −16.29 kcal mol^−1^; variance = 89.65 kcal^2^ mol^−2^) were obtained for the oxygen atom of the carbonyl function (O_7_ = −41.74 kcal mol^−1^), one of the oxygens of the B ring (O_6_ = −34.71 kcal mol^−1^) and one oxygen of the D ring (O_4_ = −32.39 kcal mol^−1^). These results made it possible to identify the atoms most likely to interact with sucrose. Interestingly, similar trends were also shown between EGCG and cholesterol [28].

As for sucrose, the lowest EP values (global mean = −57.96 kcal mol^−1^; variance = 220.34 kcal^2^ mol^−2^) were obtained for the oxygen atom of the glycosidic bond in fructose and glucose (O_2_ = −57.96 kcal mol^−1^), one of the oxygens of the B ring (O_6_ = −55.90 kcal mol^−1^) and one oxygen of the A ring (O_10_ = −52.43 kcal mol^−1^).

##### Structures and Interaction Energies of Epigallocatechin Gallate–Sucrose Complexes

Based on the possible interaction sites discussed above., and the fact that the EGCG–sucrose complex seems to form stoichiometrically (2.2.1, mass spectrometry analysis), several stoichiometric complexes were considered with the aim to assess (i) the number and lengths of H-bonds between sucrose and EGCG as well as their energy by empirical approach [29] and (ii) the stability and solubility of the EGCG–sucrose complexes by calculating their interaction energy [28] and by modeling their solvation energy by use of the polarizable continuum model (PCM) [30].

**Figure 5:**
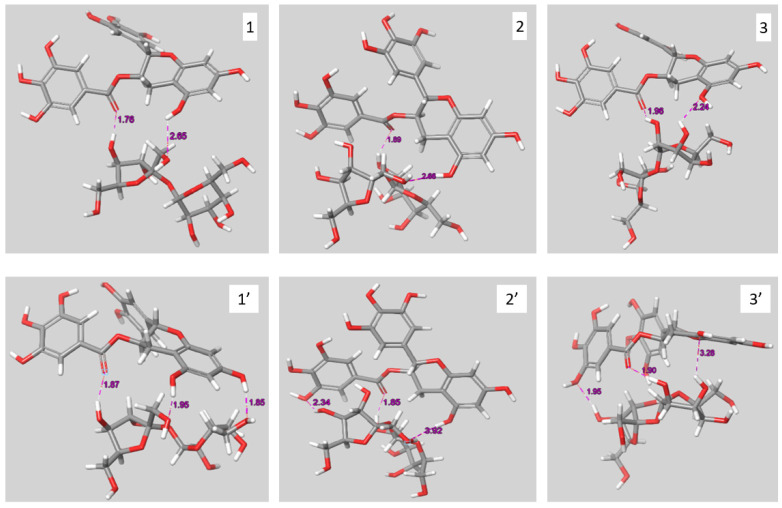
Structures of the initial (1, 2 and 3) and the geometrically optimized complexes (1′, 2′ and 3′).

The structures before (1, 2, and 3) and after (1′,2′, and 3′) geometric optimization of the three complexes, with the largest interaction energies after optimization, are reported in Figure 5. All three complexes form three hydrogen bonds where (i) the carbonyl moiety of EGCG is involved and (ii) glucose and fructose are both implicated. These results suggest that sucrose and EGCG form complexes with strong hydrogen bonds (OH—O), as has been detected with ATR-FTIR (Section 2.2.1).

All three optimized complexes exhibit strong molecular interactions both in vacuum and in the presence of water as all interaction energies are superior to 130 kJ·mol^−1^ (Table 2). These interactions result partly from the three H-bonds for complex 2 (18.8%) and complex 3 (17.6%) and to a larger extent for complex 1 (37.6%). For the three complexes, the solvation energy (Table 2) is lower than that of the compounds alone (EGCG = −108 kJ·mol^−1^; sucrose = −124 kJ·mol^−1^), in favor of a higher aqueous solubility of the complex than the separated compounds.

Thus, both experimental (ATR-FTIR and mass spectrometry) and theorical (DFT) results converge towards an effective formation of the EGCG–sucrose equimolar complex involving H-bonding and van der Waals interactions.

### 2.3. Stability of the Oral Formulation

#### 2.3.1. Evolution of EGCG’s Content in the Formulation as a Function of Temperature

EGCG in the formulation was assayed at different stability periods after months of storage and under three separate temperature conditions using ICH chambers (2–8 °C, 25 °C/60% RH and 40 °C/75% RH).

**Figure 6:**
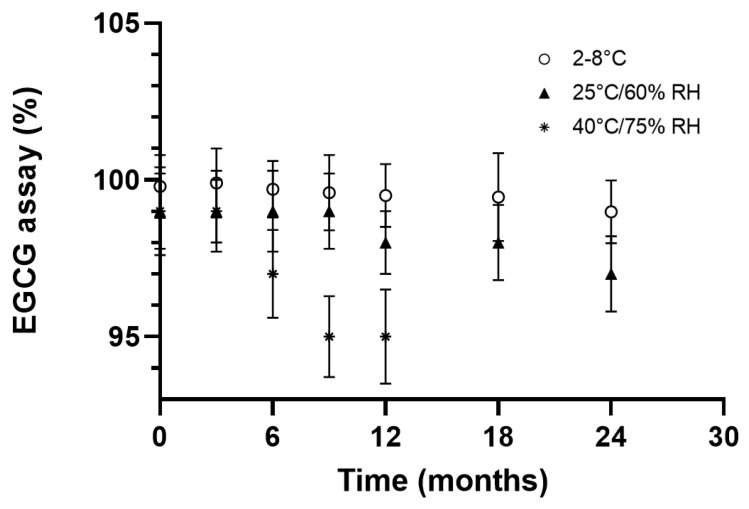
EGCG assay (%) as a function of time and storage conditions.

After a 24-month study, the formulation demonstrated satisfactory stability when stored at 2–8 °C. At 25 °C/60% RH, it is worth noticing that a decrease in the assay was observed shortly after the initial time, but beyond the three-month period, this evolution tends to reach a steady state where the product still meets specifications (Figure 6).

Under accelerated conditions, however, the chemical stability of EGCG became non-compliant, but the content still remained close to 95% after nine months of storage (Figure 6).

Compared to the literature, these outcomes seem to indicate a considerable progress in terms of stability, thus making it possible to envisage pharmaceutical development without resorting to complex and less scalable technologies. Indeed, according to the available data [8,29], the stability of EGCG in water at 37 °C depends on the concentration, but can hardly exceed a half-life of 4 h [8,29]. 

As a result, in the proposed formulation, although EGCG is formulated in an aqueous solution, the stability study data clearly demonstrate that the present formulation is suitable for use in the clinical trial, with a shelf life such that manufacturing turnaround, storage, and supply can be managed smoothly.

#### 2.3.2. Investigation on Detected Degradation Products

In these stability studies, we found that the degradation profiles differed depending on the storage temperature.

**Figure 7:**
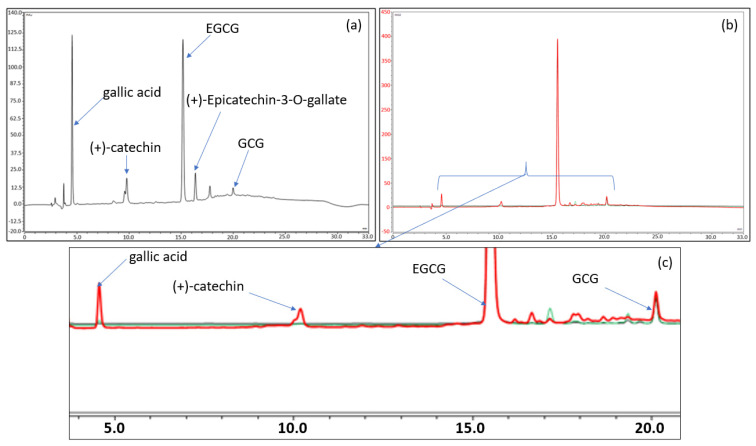
(**a**) Chromatogram of the EGCG formulation stored for 17 days at 80 °C. (**b**,**c**) Total and enlarged chromatogram of the EGCG formulation stored 6 months at 4 °C (in black), at 25 °C (in green), and at 40 °C (in red). The chromatograms have been normalized on the EGCG peak to compare the chromatographic profiles. The chromatograms of 6 months at 4 °C (in black) and at 25 °C (in green) are almost indistinguishable from each other as the drug products contain similar amounts of degradation products.

Further analyses were then carried out to shed light on what might have caused the observed variations. Thus, the most intense degradation product related to each temperature condition was identified using liquid chromatography coupled to multistage high-resolution mass spectrometry (LC-HRMS^n^), as detailed in the Supplementary Material (SM). The exact mass of these products was then compared to the different theoretical masses of the catechins and dimer constituents of green tea. It was then found—as shown in the chromatograms in Figure 7—that a small quantity of the epimerization product (GCG) is present, with slight increases over time, when the formulation is stored at 2–8 °C or 25 °C. However, no dimer and/or quinone derivatives were detected under these conditions.

At higher temperatures (40 and 80 °C), the decrease in EGCG is more rapid, but this would be due more to hydrolysis in connection with the appearance of gallic acid and still epimerization than to oxidation and/or autoxidation (Figure 7 and Supplementary Material). It is indeed likely that with a pH below 4.5, the formulation would protect EGCG more against oxidation but not against hydrolysis. A lower pH would have paved the way to thermolysis owing to the presence of the ester function.

On the other hand, the improved solubility of EGCG, allowing to obtain a concentrated solution of the active ingredient by complexation with sucrose, must have favored the epimerization process to the detriment of autoxidation and/or oxidation processes. This last finding is perfectly consistent with studies that have already reported this phenomenon [8]. 

Finally, the presence of citric acid may also have helped to combat the oxidation caused by the residual presence of metal ions, because, according to Shpigelman et al., the combination of metal complexing agents significantly helps to reduce the oxidation of EGCG [31].

It is probably the combination of all these factors within the formulation that explains the excellent stability of EGCG in its product throughout the stability studies conducted.

### 2.4. Bioavailability of the Formulation in Comparison to EGCG Solid Dosage Form (EGCG in Hard-Gel Capsule)

While the quality performance of the formulation was demonstrated by the long-term stability of this product developed for clinical trials, it is not immediately clear that it has satisfactory biopharmaceutical properties after oral administration. Indeed, one of the main causes of the low bioavailability of EGCG is its extensive degradation in the gastrointestinal tract [10], and the fact that EGCG is already in a solution may even make the situation worse. For instance, when EGCG was dissolved in normal saline, the oral bioavailability of EGCG in a freely moving rat was found to be about 4.95% [32].

To assess the performance of the formulation in terms of bioavailability, 27 mg·kg^−1^ of EGCG in the formulation was administered per os (by gavage) to rats (n = 3) and the outcome was compared to that obtained using capsules containing EGCG powder (n = 3), the quantitative composition of which had been adjusted so as to afford 27 mg·kg^−1^ of EGCG. It should be noted that the capsules used for administration to rats were of a size suitable for this type of experiment. Cmax and the total area under curves (AUC_t_) were determined for each group. 

**Figure 8:**
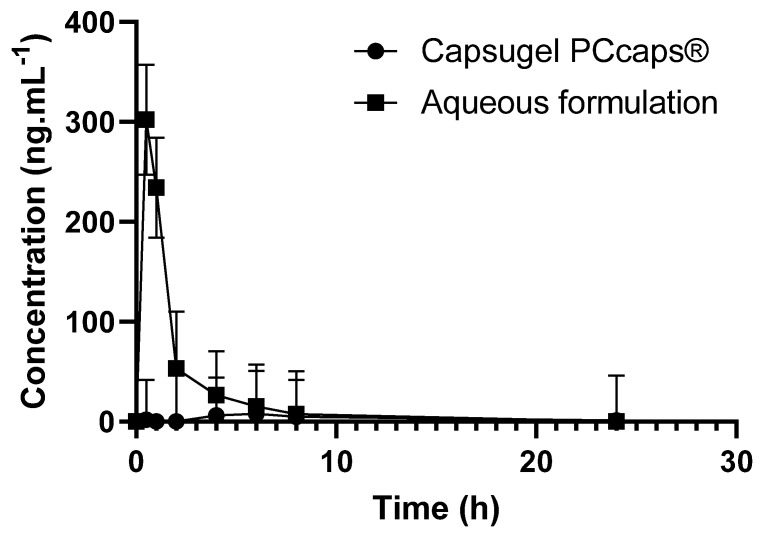
EGCG concentration as a function of time and of the formulation.

The data presented in Figure 8 show the potential value of the developed formulation as Cmax (302.4 ng·mL^−1^) and AUC_t_ (497 ng·mL^−1^·h) are, respectively, about 38 and 15 higher than when using Capsugel PCcaps^®^ containing the same amount of EGCG (Cmax = 7.9 ng·mL^−1^; AUC_t_ = 33.8 ng·mL^−1^·h). Based on the two last results where EGCG is detected in the plasma, the half-life of EGCG provided by the aqueous formulation and by the capsules is of 2.0 h and 2.8 h, respectively.

Based on these results, the formulation greatly enhances the bioavailability of epigallocatechin gallate. As depicted above, this result may be explained by the increase of EGCG in vivo stability thanks to the complex formation. The high concentration of EGCG in solution may also have contributed to the high increase in bioavailability, considering the fact that the aqueous solubility of drugs is often predictive of their bioavailability [33]. Finally, the observed soaring of bioavailability may be the consequence of an increase in EGCG in the gastrointestinal tract, as tea polyphenols are known to inhibit the activity of the efflux protein P-GP [34]. This activity of EGCG on P-GP may have led to positive feedback triggered by an increase in EGCG in the gastrointestinal tract and resulted in the soaring of the bioavailability. 

## 3. Materials and Methods

### 3.1. Materials and Reagents

EGCG was purchased from Sunfull Bio-tech Co., Ltd. All the excipients used were of pharmaceutical grade and purchased from COOPER (Melun, France). The reagents were purchased from Sigma-Aldrich. The solvents of analytical grade were obtained from Merck (Fontenay-sous-bois, France). Capsugel PCcaps^®^ (very small gelatin capsules that are ideal for oral delivery of the neat active in pre-clinical animal studies) were purchased from Lonza (Bâle, Switzerland).

### 3.2. Manufacturing Process

A pyrex glass vessel was filled with water to a volume corresponding to 1/3 the final volume of the solution for injection. Then, appropriate quantities of EGCG, citric acid, glucose, sucrose, and cola flavor were added according to Table 1 and mixed into the water until complete dissolution was obtained. After that, water for injection was added to reach the final volume. The solution was then filtered using a 1 m Millipore^®^ filtration cartridge and stocked in a stainless steel tank with a nitrogen inlet to make the solution and the headspace of the container inert. The solution was filled into a 250 mL multiple-dose amber glass bottle. The filled bottle was closed with a cap with a polyethylene seal.

### 3.3. Long-Term and Accelerated Stability Protocol

A batch consisting of 3 unit doses was prepared according to the formulation of unit dose of 250 mL mentioned in Table 1 and packed in 250 mL-multiple-dose amber glass bottles (Type III). The bottles were stored under three conditions: 2–8 °C, 25 °C/60% RH, and 40 °C/75% RH. Samples were withdrawn every 3 months during the first year following the preparation and every 6 months during the second year. The samples were diluted to one hundredth in distilled water prior to LC-UV analysis.

### 3.4. Analytical Conditions

#### 3.4.1. Infrared Conditions

The experiments were performed on a Perkin-Elmer Spectrum BX FT-IR system. The spectra of the samples were recorded at room temperature in the wavenumber range of 400–4000 cm^−1^ using an ATR cell.

#### 3.4.2. Mass Spectrometry Conditions

Mass spectrometry analysis was performed by infusing solutions by syringe or by using the chromatographic conditions described in 3.4.3 to analyze the solutions with an LTQ-Orbitrap Velos Pro system (Thermo Fisher Scientific, Waltham, MA, USA). Analyses were carried out in negative ion mode (ESI^-^) as per the following conditions.

When infusing the solutions directly in the mass spectrometer, the source voltage was set at 3.4 kV, and the source and the capillary temperatures were fixed at 53.7 °C and 300 °C, respectively. Normalized collision was set at 25 for fragmentation studies. When the solutions were analyzed under LC-HRMS^n^ conditions, the source voltage was set at 3.4 kV, and the source and the capillary temperatures were fixed at 300 °C and 350 °C, respectively. Sheath gas and auxiliary gas nitrogen flows were set at 35 and 15 arbitrary units, respectively. The S-lens was set at 60%. Normalized collision was set at 35 for high-resolution fragmentation studies. The mass range of 100–1200 amu was used for preliminary LC-HRMS studies, and of 50–600 amu for LC-HRMS^n^ studies. The MS data were processed using Xcalibur^®^ software (version 2.2 SP 1.48).

#### 3.4.3. Chromatographic Conditions

The assay of EGCG was achieved by comparing the response of a sample solution with the response of EGCG reference standard solution prepared at a similar nominal concentration and analyzed in the same way. The sample and reference standard solutions were analyzed by gradient reversed-phase LC-UV (detection wavelength set at 275 nm). Injection volume was 20 µL. Separations were performed with the VKR5 C18 (250 m × 4.6.0 m, 5 mm particle size) column purchased from Interchim^®^ using mobile phase A (5/95/0.07 *v*/*v*/*v* acetonitrile/water/acetic acid) and mobile phase B (50/50/0.05 *v*/*v*/*v* acetonitrile/water/acetic acid) and following the gradient program depicted in Table 3. The flow rate and column temperature were 1 mL·min^−1^ and 32 °C, respectively.

### 3.5. Computational

Jaguar [35] and Maestro (Maestro, Schrödinger, LLC, New York, NY, USA, 2019) programs of Schrödinger Suite 2019−1 were used for the preparation and visualization of the results of EGCG, sucrose, and the EGCG–sucrose complex. For geometry optimization, a B3LYP exchange-correlation functional with the D3 a posteriori correction [36] was chosen, together with the 6-31G++ basis set for all atoms. The energies of the molecules in water and the complexes were calculated using the conductor-like polarizable continuum model (CPCM). Interaction energies were calculated as the difference between the energies of the complexes and those of the corresponding monomers.

### 3.6. Bioavailability Studies

Because the volume administered was very small (i.e., below 300 µL), the choice of the dose was adjusted to minimize the variability of the volume to be administered, even if the dose tested is larger than that administered to humans. This is also why the rats chosen for the study also had to weigh more than 300 g to allow for a higher volume administration.

Six male Sprague-Dawley rats weighing at least of 300 g were used. The protocol was approved by the Animal Welfare Ethical Review Body of Avogadro LS on 15 January 2019. The project was approved by the French ministry “ministère de l’enseignement supérieur et de la recherche” (APAFIS#10221-2017061411259856 V2, approved on 15 November 2017). The animals had free access to food and water during the experiment.

The animals were supplied by Janvier Labs. The analytical test was based on a published method [37]. The tested conditions and plasma sampling times are reported in Table 4. The collection was performed in the sinus retro-orbital using a capillary tube. The volume of the blood collected ranged between 0.500 and 0.300 mL minimum per time point, and the anticoagulant was lithium heparin. The blood samples were centrifuged at 2500 rpm at around 10 °C, and the plasma was then removed and placed into labeled polypropylene tubes. Individual plasma samples were stored frozen (−20 °C ± 5 °C) until analysis.

## 4. Conclusions

Conditions to obtain a stable and concentrated EGCG solution appropriate for clinical use were proposed. The mechanism through which the solubility and the stability of EGCG are increased was studied using a combination of experimental and theorical studies. 

Formation of the EGCG–sucrose complex highly increased the stability of EGCG in a formulation insofar as the proposed formulation is stable at least up to 24 months at 2–8 °C or 25 °C. Furthermore, the formation of unwanted (auto)oxidation products, which may jeopardize the drug product’s safety, is reduced in the proposed formulation. Finally, the complex formation resulted in a substantial increase in the absorbed quantity of EGCG.

The approach proposed in this article may be used in other contexts as the characterization of the molecular interactions specific to an active ingredient can be useful in designing formulations to increase the solubility, stability, and bioavailability of the considered ingredient.

## 5. Patents

A patent resulted from the work reported in this manuscript [12].

## Figures and Tables

**Table 1:** Composition of the solution.

	Percentage Formula (% *w*/*w*)
EGCG	2.68
Citric acid	0.05
Glucose	23.4
Sucrose	10.0
Cola flavor	0.6
Water for injection	ad 100%

**Table 2:** Interaction energies (kJ·mol^−1^) in vacuum and water, length of H-bonds (A) and associated energy (kJ·mol^−1^), and solvation energy (kJ·mol^−1^).

Complex	Interaction Energy (Vacuum)	Interaction Energy (Water)	R (O-----H)1	R (O-----H)2	R (O-----H)3	Interaction Energy due to H-Bonding (%)	Solvation Energy
1	−170.24536	−130.99289	1.85 (23)	1.87 (22)	1.95 (19)	37.6	−140.2895
2	−192.98007	−139.20560	1.85 (23)	2.34 (11)	3.92 (2.3)	18.8	−137.1217
3	−250.44187	−196.03839	1.9 (21)	1.95 (19)	3.28 (4.0)	17.6	−137.7863

**Table 3:** Chromatographic program.

Time (min)	A (%)	B (%)
0	90	10
10	80	20
16	60	40
20	50	50
25	50	50
27	60	40
30	90	10
33	90	10
45	90	10

**Table 4:** Conditions of the bioavailability studies.

Reference Compounds	Route of Administration	Vehicle	Concentration (mg/mL)	Volume of Administration (mL/kg)	Selected Dose (mg/kg)	Plasma Sampling Times
EGCG	PO	Administration via 1 PCcaps^®^	-	-	27	30 min, 1 h, 2 h, 4 h, 6 h, 8 h, 24 h
EGCG	PO	Formulation described in Table 1	27	1	27	30 min, 1 h, 2 h, 4 h, 6 h, 8 h, 24 h

## Data Availability

The data is contained in the article and Supplementary Materials.

## Supplementary Materials

The following are available online at https://www.mdpi.com/article/10.3390/ph14121242/s1. Figure S1: (a): LC-HRMS spectrum obtained at the retention time of the main degradation product; (b): LC-HRMS^2^ spectrum of the main product ion detected in the LC-HRMS spectrum (*m*/*z* = 169).

## References

[1] Kim H.-S., Quon M.J., Kim J. (2014). New insights into the mechanisms of polyphenols beyond antioxidant properties; lessons from the green tea polyphenol, epigallocatechin 3-gallate. Redox Biol..

[2] Du G.-J., Zhang Z., Wen X.-D., Yu C., Calway T., Yuan C.-S., Wang C.-Z. (2012). Epigallocatechin Gallate (EGCG) Is the Most Effective Cancer Chemopreventive Polyphenol in Green Tea. Nutrients.

[3] Higdon J.V., Frei B. (2003). Tea Catechins and Polyphenols: Health Effects, Metabolism, and Antioxidant Functions. Crit. Rev. Food Sci. Nutr..

[4] BSingh N., Shankar S., Srivastava R.K. (2011). Green tea catechin, epigallocatechin-3-gallate (EGCG): Mechanisms, perspectives and clinical applications. Biochem. Pharmacol..

[5] Chow H.-H.S., Hakim I.A., Vining D.R., Crowell J.A., Ranger-Moore J., Chew W.M., Celaya C.A., Rodney S.R., Hara Y., Alberts D.S. (2005). Effects of Dosing Condition on the Oral Bioavailability of Green Tea Catechins after Single-Dose Administration of Polyphenon E in Healthy Individuals. Clin. Cancer Res..

[6] Xu Y.-Q., Yu P., Zhou W. (2019). Combined effect of pH and temperature on the stability and antioxidant capacity of epigallocatechin gallate (EGCG) in aqueous system. J. Food Eng..

[7] Zeng J., Xu H., Cai Y., Xuan Y., Liu J., Gao Y., Luan Q. (2018). The Effect of Ultrasound, Oxygen and Sunlight on the Stability of (−)-Epigallocatechin Gallate. Molecules.

[8] Krupkova O., Ferguson S.J., Wuertz-Kozak K. (2016). Stability of (−)-epigallocatechin gallate and its activity in liquid formulations and delivery systems. J. Nutr. Biochem..

[9] Sang S., Lambert J.D., Ho C.-T., Yang C.S. (2011). The chemistry and biotransformation of tea constituents. Pharmacol. Res..

[10] Cai Z.-Y., Li X.-M., Liang J.-P., Xiang L.-P., Wang K.-R., Shi Y.-L., Yang R., Shi M., Ye J.-H., Lu J.-L. (2018). Bioavailability of Tea Catechins and Its Improvement. Molecules.

[11] Paliwal R., Babu R.J., Palakurthi S. (2014). Nanomedicine Scale-up Technologies: Feasibilities and Challenges. AAPS PharmSciTech.

[12] Do B., Paul M., Astier A. (2020). Epigallocathechin Gallate Solution, US20200375939A1. https://patents.google.com/patent/US20200375939A1/en?q=EPIGALLOCATHECHIN+GALLATE+SOLUTION&oq=EPIGALLOCATHECHIN+GALLATE+SOLUTION.

[13] Quideau S., Deffieux D., Douat-Casassus C., Pouységu L. (2011). Plant Polyphenols: Chemical Properties, Biological Activities, and Synthesis. Angew. Chem. Int. Ed..

[14] Henning S.M., Choo J.J., Heber D. (2008). Nongallated Compared with Gallated Flavan-3-ols in Green and Black Tea Are More Bioavailable. J. Nutr..

[15] Mochizuki M., Yamazaki S., Kano K., Ikeda T. (2002). Kinetic analysis and mechanistic aspects of autoxidation of catechins. Biochim. Biophys. Acta BBA-Gen. Subj..

[16] Hagerman A.E., Dean R.T., Davies M.J. (2003). Radical chemistry of epigallocatechin gallate and its relevance to protein damage. Arch. Biochem. Biophys..

[17] Wang R., Zhou W., Jiang X. (2008). Reaction Kinetics of Degradation and Epimerization of Epigallocatechin Gallate (EGCG) in Aqueous System over a Wide Temperature Range. J. Agric. Food Chem..

[18] Suzuki M., Sano M., Yoshida R., Degawa M., Miyase T., Maeda-Yamamoto M. (2003). Epimerization of Tea Catechins and O-Methylated Derivatives of (−)-Epigallocatechin-3-O-gallate: Relationship between Epimerization and Chemical Structure. J. Agric. Food Chem..

[19] Donaldson M., Goodchild J.H., Epstein J.B. (2015). Sugar content, cariogenicity, and dental concerns with commonly used medications. J. Am. Dent. Assoc..

[20] Pal Y., Deb P.K., Bandopadhyay S., Bandyopadhyay N., Tekade R.K. (2018). Role of Physicochemical Parameters on Drug Absorption and Their Implications in Pharmaceutical Product Development. Dosage Form Design Considerations.

[21] Balbani A.P.S., Stelzer L.B., Montovani J.C. (2006). Pharmaceutical excipients and the information on drug labels. Braz. J. Otorhinolaryngol..

[22] Ali M.A., Rahman M.S., Roy R., Gambill P., Raynie D.E., Halim M.A. (2021). Structure Elucidation of Menthol-Based Deep Eutectic Solvent using Experimental and Computational Techniques. J. Phys. Chem. A.

[23] Dotsikas Y., Loukas Y.L. (2003). Efficient determination and evaluation of model cyclodextrin complex binding constants by electrospray mass spectrometry. J. Am. Soc. Mass Spectrom..

[24] Kwon S., Lee W., Shin H.-J., Yoon S., Kim Y., Kim Y.-J., Lee K., Lee S. (2009). Characterization of cyclodextrin complexes of camostat mesylate by ESI mass spectrometry and NMR spectroscopy. J. Mol. Struct..

[25] Li H., Zhou J., Tang F., Yuan G. (2006). Investigation of noncovalent complexes between β-cyclodextrin and polyamide acids containing *N*-methylpyrrole and *N*-methylimidazole by electrospray ionization mass spectrometry. J. Am. Soc. Mass Spectrom..

[26] Murray J.S., Seminario J.M., Politzer P., Sjoberg P. (1990). Average local ionization energies computed on the surfaces of some strained molecules. Int. J. Quantum Chem..

[27] Armaković S., Armaković S.J., Šetrajčić J.P., Šetrajčić I.J. (2012). Active components of frequently used β-blockers from the aspect of computational study. J. Mol. Model..

[28] Zheng K., Guo K., Xu J., Liu W., Chen J., Xu C., Chen L. (2020). Study on the interaction between catechin and cholesterol by the density functional theory. Open Chem..

[29] Rozenberg M., Loewenschuss A., Marcus Y. (2000). An empirical correlation between stretching vibration redshift and hydrogen bond length. Phys. Chem. Chem. Phys..

[30] Cossi M., Barone V., Cammi R., Tomasi J. (1996). Ab initio study of solvated molecules: A new implementation of the polarizable continuum model. Chem. Phys. Lett..

[31] Shpigelman A., Zisapel A., Cohen Y., Livney Y.D. (2013). Mechanisms of saccharide protection against epigallocatechin-3-gallate deterioration in aqueous solutions. Food Chem..

[32] Lin L.-C., Wang M.-N., Tseng T.-Y., Sung J.S., Tsai T.-H. (2007). Pharmacokinetics of (−)-Epigallocatechin-3-gallate in Conscious and Freely Moving Rats and Its Brain Regional Distribution. J. Agric. Food Chem..

[33] Savjani K.T., Gajjar A.K., Savjani J.K. (2012). Drug Solubility: Importance and Enhancement Techniques. ISRN Pharm..

[34] Jodoin J., Demeule M., Béliveau R. (2002). Inhibition of the multidrug resistance P-glycoprotein activity by green tea polyphenols. Biochim. Biophys. Acta BBA-Mol. Cell Res..

[35] Bochevarov A.D., Harder E., Hughes T.F., Greenwood J.R., Braden D.A., Philipp D.M., Rinaldo D., Halls M.D., Zhang J., Friesner R.A. (2013). Jaguar: A high-performance quantum chemistry software program with strengths in life and materials sciences. Int. J. Quantum Chem..

[36] Theilacker K., Arbuznikov A.V., Bahmann H., Kaupp M. (2011). Evaluation of a Combination of Local Hybrid Functionals with DFT-D3 Corrections for the Calculation of Thermochemical and Kinetic Data. J. Phys. Chem. A.

[37] Mata-Bilbao M.D., Andrés-Lacueva C., Roura E., Jáuregui O., Torre C., Lamuela-Raventós R.M. (2007). A New LC/MS/MS Rapid and Sensitive Method for the Determination of Green Tea Catechins and their Metabolites in Biological Samples. J. Agric. Food Chem..

