# Supplementary material for: Simple Approach to Enhance Green Tea Epigallocatechin Gallate Stability in Aqueous Solutions and Bioavailability: Experimental and Theoretical Characterizations
Source: Pharmaceuticals (Basel). 2021 Nov 30;14(12):1242. doi: 10.3390/ph14121242 (PMC8706847; doi:10.3390/ph14121242)
Supplement: Supplementary file 1 [file pharmaceuticals-14-01242-s001.zip › pharmaceuticals-1458143-supplementary.pdf]

Supplementary information to “Simple approach to enhance green tea epigallocatechin gallate stability in aqueous solutions and bioavailability: experimental and theoretical characterizations”

Philippe-Henri Secretan [1,*,†], Olivier Thirion [2,†], Hassane Sadou Yayé [3], Thibaud Damy [4], Alain Astier [2], Muriel Paul [2,5] and Bernard Do [1,2]

1 Université Paris-Saclay, Matériaux et santé, 92296 Châtenay-Malabry, France; bernard.do@aphp.fr
2 Department of Pharmacy, Hôpitaux Universitaires Henri Mondor, AP-HP, 94000, Créteil, France; olivier.thirion@aphp.fr (O.T.); prof.astier@gmail.com (A.A.); muriel.paul@aphp.fr (M.P.)
3 Department of Pharmacy, Hôpitaux Universitaires Pitié-Salpêtrière, AP-HP, 75013, Paris, France; hassane.sadou-yaye@aphp.fr
4 Département de Cardiologie et des maladies Vasculaires , Hôpitaux Universitaires Henri Mondor, AP-HP, 94000, Créteil, France; thibaud.damy@aphp.fr
5 EpidermE, Université Paris Est Creteil, 94010 Creteil, France
* Correspondence: phsecretan@yahoo.fr
† The first 2 authors contributed equally to this study and are therefore considered as first authors.

Structural elucidation of gallic acid

Regarding the mainly detected degradation product (Figure 7, retention time = about 4.5 min), its accurate mass in LC-HRMS (Figure S1 (a); accurate mass = 169.0145) corresponded to the molecular formula $C_7H_5O_5^-$ (mass error=4.98 ppm) which could be assigned to gallic acid whose structure is reported in Figure S1 (a). This hypothesis was confirmed by the base peak (Figure S1 (b); accurate mass=125.02478) obtained when submitting m/z = 169 to collision induced dissociation (LC-HRMS^2). Indeed, its accurate mass corresponds to the loss of the carboxylic acid moiety (mass error= 7.21 ppm). As far as the other detected compounds, they have been identified as (+)-catechin, (+)-Epicatechin-3-O-gallate and GCG based upon their respective accurate masses.

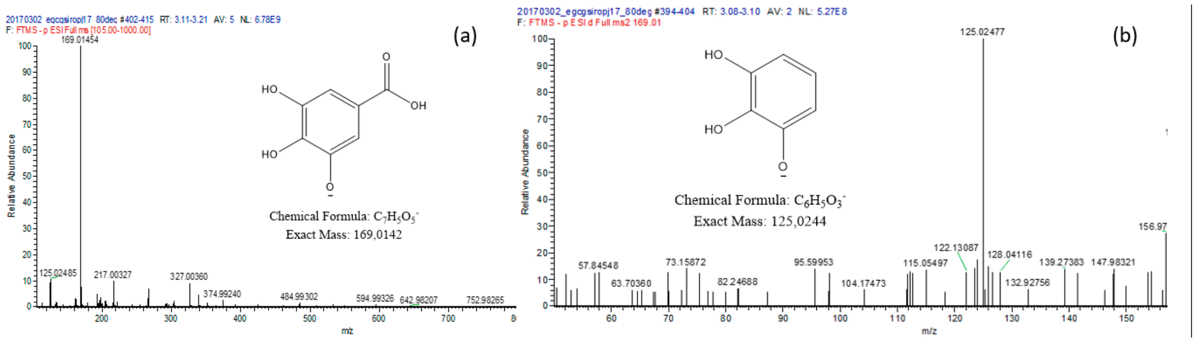

Figure S1. (a) : LC-HRMS spectrum obtained at the retention time of the main degradation product ; (b): LC-HRMS^2 spectrum of the main product ion detected in the LC-HRMS spectrum (m/z=169)
